# Bundle formation of sperm: Influence of environmental factors

**DOI:** 10.3389/fendo.2022.957684

**Published:** 2022-10-10

**Authors:** Paula Morcillo i Soler, Carlos Hidalgo, Zoltán Fekete, Laszlo Zalanyi, Islam S. M. Khalil, Marc Yeste, Veronika Magdanz

**Affiliations:** ^1^ Smart Nanobiodevices Group, Institute for Bioengineering of Catalonia, Barcelona, Spain; ^2^ Centro de Biotecnológia Animal SERIDA-DEVA-GIJON, Gijón, Spain; ^3^ ONGO Vettech Ltd., Martonvásár, Hungary; ^4^ Faculty of Information Technology & Bionics, Pazmany Peter Catholic University, Budapest, Hungary; ^5^ Department of Computational Sciences, Wigner Research Centre for Physics, Budapest, Hungary; ^6^ Department of Biomechanical Engineering, University of Twente, Enschede, Netherlands; ^7^ University of Girona, Girona, Spain; ^8^ Catalan Institution for Research and Advanced Studies (ICREA), Barcelona, Spain; ^9^ Systems Design Engineering, University of Waterloo, Waterloo, ON, Canada

**Keywords:** spermatozoa, cooperative behaviour, bundling, cell-cell interaction, sperm migration, sperm selection, sperm competition

## Abstract

Cooperative behaviour of sperm is one of the mechanisms that plays a role in sperm competition. It has been observed in several species that spermatozoa interact with each other to form agglomerates or bundles. In this study, we investigate the effect of physical and biochemical factors that will most likely promote bundle formation in bull sperm. These factors include fluid viscosity, swim-up process, post-thaw incubation time and media additives which promote capacitation. While viscosity does not seem to influence the degree of sperm bundling, swim-up, post-thaw migration time and suppressed capacitation increase the occurrence of sperm bundles. This leads to the conclusion that sperm bundling is a result of hydrodynamic and adhesive interactions between the cells which occurs frequently during prolonged incubation times.

## 1 Introduction

Many guidance mechanisms help spermatozoa reach the fertilization site. Besides taxis behavior of sperm cells in response to chemicals, fluid flows, temperature gradients and surfaces, interactions between sperm cells within an ejaculate also play a rolecitepEisenbach2006. While chemotaxis is thought to play a role in short-distance guidance, temperature gradients are expected to serve as guidance over long distances (1). Interactions with surfaces and fluid flow are physical guidance mechanisms independent on the distance to the oocyte. Little is known about how sperm cells interact with each other to assist one another on the journey to the oocyte. Sperm-sperm interactions occur and form a type of cooperative behaviour.

Multi-flagellated sperm and bundling of sperm has been found in many insects (beetles, flies, termites, ants) and remains an interesting phenomenon to be studied regarding its role in sperm cooperation and competition (2, 3). Especially in murine or rodent sperm with a flat, hook-like sperm head, bundling has been observed (4–7). Known as sperm trains, these spermatozoa attach by their heads which results in collective swimming with increased speed. It was reported that some rodent sperm preferentially cooperate with closely related sperm, hinting to an adaptation that could be driven by sperm competition (4). In Drosophila, the tight bundling of giant sperm enables the concerted crawling and forward motion of the sperm mass (8). In ant sperm large bundles of sperm can be observed that are frequently ejaculated as such, held together by glycoproteins (2). Overall, this cooperative behaviour of sperm is expected to help the sperm protect each other from the surroundings (e.g. during sperm storage), align with each other (in case of giant sperm), and guide each other in the right direction as well as concerted motion to save energy during prolonged migration time.

Viscoelasticity of the medium is known to promote the collective behavior of sperm, displayed by the formation of dynamic groups of sperm swimming together (9). Hence, cell-to-cell interactions are likely to play a promoting role in successful sperm migration to the fertilization site; yet, only a few studies have investigated this phenomenon in mammalian sperm outside the evolutionary context (10). Further, most fertility studies focus on behaviour of single cells, whereas investigations of collective cell behaviour have not been the main focus, despite their relevance in natural conditions. It is well known that millions of sperm cells start off their journey through the reproductive tract, and even though only one sperm cell successfully fertilizes the oocyte, cell-cell interactions play a major role in sperm migration. The hydrodynamic interactions between spermatozoa have been studied previously and it has been shown that their flagella can synchronize their beating patterns in order to enhance their swimming motion, characterized by superior swimming velocity over single sperm (11). Further, a geometrical model has demonstrated that the flat, ellipsoid heads of spermatozoa easily pair up into bundles due to adhesive regions on their acrosomal areas (6). Cooperation between sperm is a behaviour that assists them on their way to the fertilization site and enhances their chance to reach the oocyte. It is thought that sperm cooperative motion evolved to improve motility under high selection pressure in a competitive context (12).

To understand the formation of bundles, the maturation process of spermatozoa has to be considered, as it brings along many biochemical changes. In mammals, ejaculated spermatozoa must go through a process called capacitation before fertilizing the egg. Capacitated sperm show physiological changes such as hyperactivation (13), acrosomal reaction and increased membrane fluidity, as well as biochemical changes that include the increase of tyrosine phosphorylation of sperm proteins (14, 15), rises in intracellular pH and Ca^2+^ concentration (16), membrane hyperpolarization and cholesterol efflux (15, 17, 18). The intracellular increase of concentration of the second messenger Ca^2+^ and tyrosine phosphorylation of sperm flagellar proteins trigger hyperactive sperm motion. This leads to a change in the motility pattern due to increasing flagellar bend asymmetry and swimming path curvature (13). These are important changes that are able to regulate cell-cell interactions and adhesion. Spermatozoa undergo these maturation processes *in vivo* in the female reproductive tract by the exposure to oviductal fluid and soluble factors (19). Similarly, capacitation can be induced *in vitro* by supplementing the media with a variety of sperm capacitating agents, such as bicarbonate (20), progesterone (21, 22), heparin (22, 23) and albumin (24, 25). In effect, bovine serum albumin (BSA) has been reported to induce Ca^2+^ influx in sperm cells, although the molecular mechanisms underlying such influx are not well understood (25). Effects of BSA are going to be studied in this study by using Fluo-3/PI staining and flow cytometry. Fluo-3, to measure intracellular calcium concentrations and propidium iodide (PI) to evaluate sperm viability (25).

In this study, we take an experimental approach to investigate how physical and biochemical factors induce formation of sperm bundles *in vitro*. We focus on migration time, viscosity, before versus after swim-up and capacitation. To mimic the sperm migration to reach the oocyte we use the swim-up technique, a simple selection method based on the active movement of the spermatozoa from the bottom pellet into an overlayered medium, accumulating the most motile cells in the upper fraction (26, 27), avoiding centrifugation and prewashing steps to decrease sperm manipulation. We perform a swim-up procedure and investigate sperm from the upper fraction at different post-thaw-migration times from 0 h (before swim-up) up to 3.5 h in two different media (DMEM medium and DMEM + BSA medium, to induce capacitation). We also investigate sperm bundling in media with different levels of viscosity by addition of methylcellulose (0.1 - 0.6%), an inert chemical that increases the viscosity of the medium (See supporting information). Sperm swim through a variety of fluids with elevated viscosity on their way to the fertilization site. Viscosity is thought to act as a selection mechanism in the reproductive tract. It has been demonstrated that a change in viscosity alters flagella beating parameters and thus their motility (28). Hence, we investigate sperm bundle formation in different levels of viscosity up to 80 mPas. This range was chosen based on previous studies that investigate sperm motion in *in vivo* mimicking conditions, taking into account the viscosities of reproductive tract fluids (29–31).

## 2 Materials and methods

### 2.1 Preparation of sperm media

The medium was based on non-supplemented high glucose Dulbecco’s Modified Eagle Medium (ThermoFisher) containing 4.5 g/L D-Glucose and L-Glutamine. Capacitation medium was prepared by adding 6 mg/ml of Bovine Serum Albumin (Sigma Aldrich) to DMEM medium. Different viscosities media were produced by adding methylcellulose (M0512, Sigma Aldrich), from 0.2%, 0.4%, 0.6% to 1.2%, subsequently diluted to reach final viscosities (0%, 0.1%, 0.2%, 0.4% and 0.6% Methyl cellulose) to observe the preparation by microscope. Viscosity was measured by a rheological analysis (Anton Paar, cone-plate rheometer) with a shear rate from 0.01 to 1000 s-1, at 25°C and 37°C. DMEM medium had a viscosity of 0.93 mPa·s, DMEM + 0.1%, DMEM + 0.2%, DMEM + 0.4% and DMEM + 0.6% had a viscosity of 2.15, 4.26, 13.5, 20.3 mPa·s, respectively. Adding 6 mg/ml BSA to DMEM medium, increased viscosity to 2.33 mPa·s, in 0.1%, 0.2%, 0.4% and 0.6% corresponding to 4.34, 9.76, 35.91 and 79.923 mPa·s, accordingly (See **Supporting Information Figure S1**).

### 2.2 Thawing of cryopreserved bovine semen

Cryopreserved Asturian and Holstein bovine semen straws from the Animal Biotechnology Center Sèrida were used for this study. Sperm straws were thawed in 38°C water bath for 1 minute. Sperm concentration and % of total motility was measured in each post-thawing time using ONGO Vettech semen analyzer. Sperm cell concentration was on average 10^7^ cells/mL before the swim-up (0 h) with 44.70% of total motility. The total number of cells decreased as swim-up process was going on but the % of motility increased with time until 2 hours, as displayed in **Figure S4** in **Supporting Information**

Swim-Up was immediately performed layering carefully 200 *μ*L of undiluted thawed bull semen under 500 *μ*L of medium. The Eppendorf tube was placed at a 45 degree angle in the 38°C incubator for 45 min. Non swim-Up procedure was done with a 1:1 thawed sperm and medium dilution during the first 45 minutes. The same protocol was performed with both DMEM and DMEM + BSA media. Videomicroscopy was performed by mixing 5 *μ*L of sperm from the upper swim-up fraction or from any mixed part of the non-swim-up one with 5 *μ*L of different viscosity medium distributed in different times (0 h, 1 h, 1.5 h, 2 h, 2.5 h, 3 h, 3.5 h) to reach the final viscosities (0%, 0.1%, 0.2%, 0.4% and 0.6% Methyl cellulose).

### 2.3 Highspeed videomicroscopy and video analysis

Videos were acquired by high speed videomicroscopy using a NX4 camera (ITD) with Motion Studio software mounted to a Leica DMI3000 B inverted microscope with a 20x/Ph 1 objective, recording 200 frames per second for 2 seconds. Video analysis was manually conducted with ImageJ program, focusing on bundles and cells recount, % of motility, bundles velocity and the recording time after swim-Up procedure. Overall sperm motility percentage and concentration were determined with ONGO Vettech semen analyzer and 20 micron disposable glass chamber.

### 2.4 Flow cytometry

Flow cytometry was performed with Cell Lab Quanta™ SC MPL (Beckman Coulter) to evaluate sperm viability and different sperm capacitation parameters during different post-thawing times. Two vials with sperm cells were prepared after thawing 4 straws and pooling them into the bottom of each tube that contain 2 mL of DMEM medium and 2 mL of DMEM medium + BSA in the other vial. The tubes were placed at a 45 degree angle in a 38°C water bath for 45 min. Every 30 min starting after 1 h of swim-up, 30 *μ*Lwere withdrawn from the top fraction and diluted with 320 *μ*L of PBS. Samples were excited with an argon ion laser (488 nm) set at a power of 22 mW. Two optical filters were used: FL1 (green fluorescence; Dichroic/Splitter, DRLP: 550 nm, BP filter: 525 nm, detection width 505 nm-545 nm) and FL3 (red fluorescence; LP filter: 670 nm). Signals were logarithmically amplified and photomultiplier settings were adjusted to particular staining methods. Sheath flow rate was set at 4.17 *μ*l min^-1^ in all analyses, and EV and side scatter (SS) were recorded in a linear mode (inEE vs. SS dot plots) for a minimum of 10,000 events per replicate. The analyzer threshold was adjusted on the EV channel to exclude subcellular debris (particle diameter <7 *μ*m) and cell aggregates (particle diameter >12 *μ*m). Therefore, the sperm-specific events, which usually appeared in a typically L-shaped scatter profile, were positively gated on the basis of EV and SS distributions, while the others were gated out. In all cases except for the SYBR-14/PI assessment, data obtained from flow cytometry experiments were corrected according to the procedure set by Petrunkina et al. (2010) (32). Each assessment for each sample and parameter was repeated three times in independent tubes, prior to calculating the corresponding mean ± SEM.

#### 2.4.1 Sperm viability (SYBR-14/PI)

Sperm viability was assessed using the LIVE/DEAD^®^ Sperm Viability Kit (SYBR-14/PI), according to the protocol described by Garner and Johnson (1995) (33). Briefly, sperm samples were incubated at 38 °C for 10 min with SYBR-14 at a final concentration of 100 nM, and then with PI at a final concentration of 10 *μ*M for 5 min at the same temperature. Three sperm populations were identified: i. viable green-stained spermatozoa (SYBR-14+/PI-); ii. non-viable red-stained spermatozoa (SYBR-14-/PI+), and iii. non-viable spermatozoa that were stained both green and red (SYBR-14+/PI+). Non-sperm particles (debris) were found in SYBR-14-/PI- quadrant. Single-stained samples were used for setting the electronic volume (EV) gain, FL1 and FL3 PMT-voltages and for compensation of SYBR-14 spill over into the PI channel (2.45%).

#### 2.4.2 Intracellular calcium (Fluo3/PI)

Sperm samples were incubated for 10 min at 38 °C with Fluo-3-AM (final concentration: 1 *μ*M) and PI (final concentration: 12 *μ*M). A total of four sperm populations were identified: i. viable spermatozoa with low levels of intracellular calcium (Fluo-3-/PI-); ii. viable spermatozoa with high levels of intracellular calcium (Fluo-3+/PI-); iii. non-viable spermatozoa with low levels of intracellular calcium (Fluo-3-/PI+), and iv. non-viable spermatozoa with high levels of intracellular calcium (Fluo-3+/PI+). Unstained and single-stained samples were used for setting the EV-gain, FL-1 and FL-3 PMT voltages and for compensating Fluo-3 spill over into the FL3-channel (2.45%) and PI spill-over into the FL1-channel (28.72%).

### 2.5 Analysis of CTC staining with fluorescent microscopy

Chlortetracycline (CTC) staining was performed to evaluate the capacitation state of the sperm cells forming the bundles. Chlortetracycline is a fluorescent stain, which forms chelate complexes in the presence of calcium ions. It visualizes the calcium redistribution in the sperm membrane upon capacitation events in three patterns, depending on the sperm capacitation state. The “F-Pattern” shows sperm that are noncapacitated, the “B-Pattern” shows capacitated sperm with an intact acrosome, the “AR” pattern shows capacitated sperm with a reacted acrosome (see **Figure S3** in **Supporting Information**). Bovine serum albumin triggers capacitation, and therefore, the CTC assay is only performed in T-medium without BSA, despite the fact that in the presence of BSA, only few bundles were observed. Chlortetracycline hydrochloride was obtained from Sigma-Aldrich (C4881). The CTC-stock solution (pH 7.8) was prepared by adding 0.75 mM chlortetracycline, 20 mM Tris (VWR), 0.13 M NaCl (Sigma Aldrich), and 5 mM DL-cysteine (Sigma Aldrich). 10 *μ*L of the sperm suspension and 10 *μ*L of CTC-solution were carefully mixed on a microscope slide and covered with a 22 × 22 mm2 glass cover slide. After 10 - 15 min of incubation in darkness at 37°C, the stained cells were imaged at 200× total magnification and 440 nm excitation in a Thunder Leica inverted microscope.

### 2.6 Statistics

Statistical analysis was performed using RStudio software 4.1.3 version. The first analysis was done to check normality (Shapiro-Wilks test) and homogeneity of variances (Levene test) assumptions. Most of the data were found not to match normal distribution even after logarithmic transformations. Parametric data following normality and homogeneity assumptions tests were compared with ANOVA followed by Bonferroni *post-hoc* test. Non-parametric data were tested with Kruskal-Wallis test, and pair-wise comparisons were made with Wilcox test (See **Supplementary Material**). In all statistical tests, the significance level was set at P ≤ 0.05.

## 3 Results

**Figure 1:**
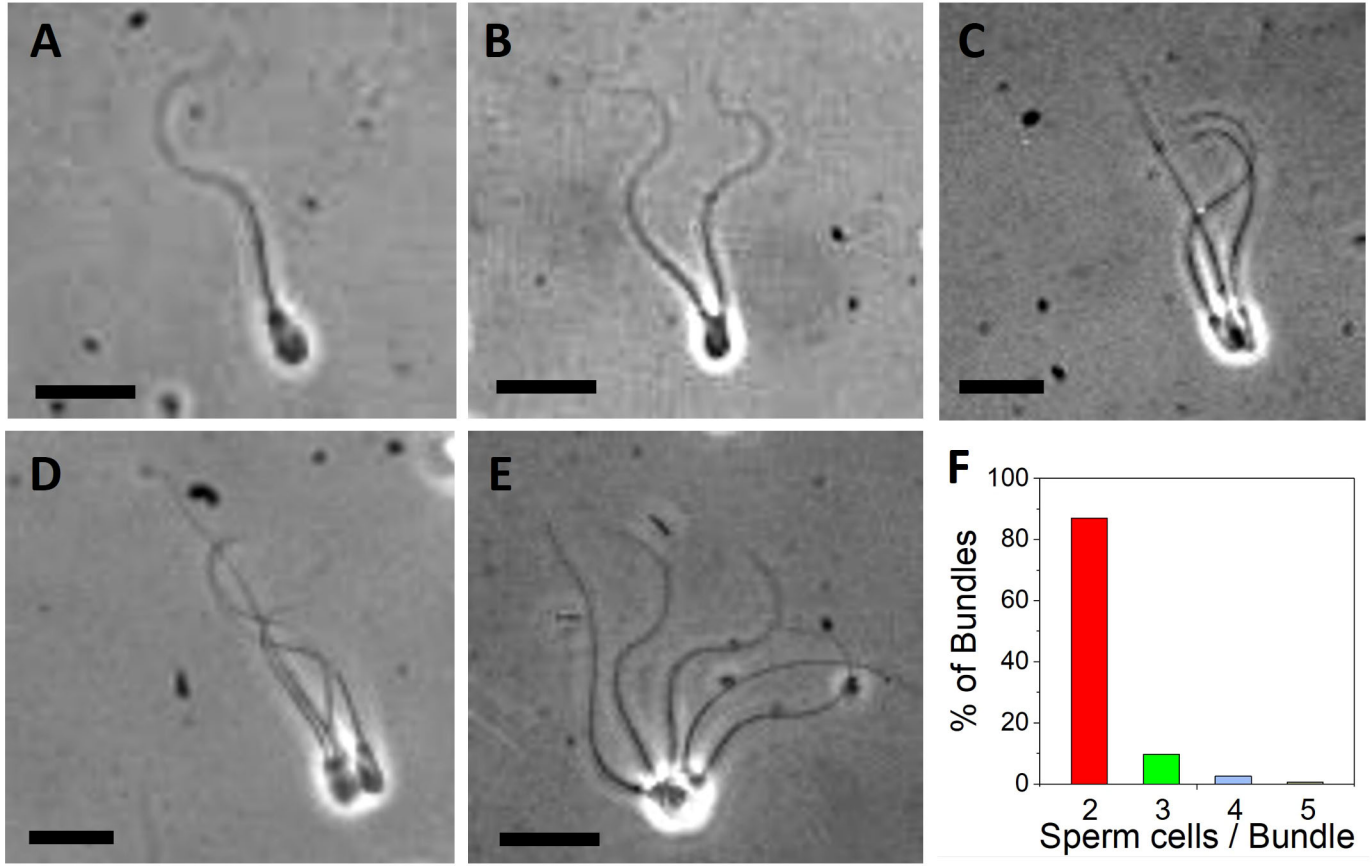
Images of bovine sperm bundles obtained from videomicroscopy. **(A)** Single sperm cell. Bundle with **(B)** 2 cells **(C)** 3 cells **(D)** 4 cells **(E)** 5 cells. **Video 1–4** show representative cases. Scale bars are 20 µm. **(F)** Distribution of observed bundles depending on the number of sperm cells per bundle (2, 3, 4 and 5 cells per bundle).

Bundle formation of cryopreserved bull spermatozoa was observed after thawing during videomicroscopy (see also videos in **Supplementary Information**). Phase contrast images of single spermatozoa (**Figure 1A**), sperm pairs (**Figure 1B**), bundles of three (**Figure 1C**), four (**Figure 1D**) and five cells (**Figure 1E**) illustrated that bull sperm tend to adhere to each other on their heads. **Figure 1A** shows a single bovine sperm cell, characterized by an increasing flagella amplitude along its tail length. When two cells bundled (**Figure 1B** as shown in **Video 1**), their flagella could display synchrony in their beating pattern. The bundle of three cells shown in **Figure 1C** and **Video 2** contains two cells that exhibit hydrodynamic synchronization, while the third cell is passive. **Figure 1D** and **Video 3** shows a bundle with four cells that are partially synchronizing. **Figure 1E** and **Video 4** shows a bundle of five cells in which the sperm heads are not aligned well, which has a negative impact on the net forward motion of the bundle. By far most bundles (over 80%) contained two cells (**Figure 1F**), while 10% of bundles contained three cells and less than 5% of bundles contained four or more cells.

### 3.1 Fluid viscosity does not alter bundle formation

**Figure 2:**
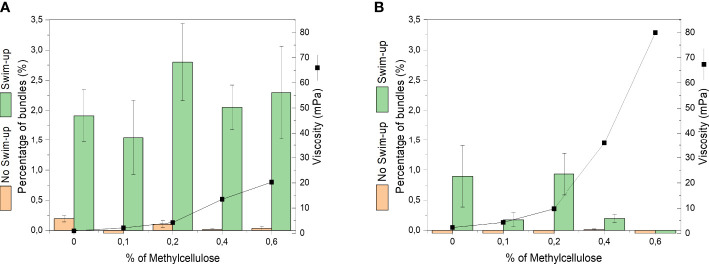
Occurrence of cell bundles as percentage of total number of bull sperm before and after swim-up in different levels of viscosity (different concentrations of methyl cellulose). Viscosities are average values at shear rate 100 1/s. **(A)** DMEM **(B)** DMEM medium + BSA medium.

We observed sperm bundling across different levels of viscosity from 1.2 mPas to 80mPas, generated by addition of methyl cellulose (MC) to the medium (see **Materials and Methods** and **Supporting Information Figure 2**). After swim-up for 45 min, sperm cells were transferred into media of different viscosity and their motility and bundling events were observed under the microscope. When plotting the percentage of sperm bundles (related to the whole population of observed sperm cells) over the viscosity, no correlation between viscosity and bundle occurrence was found (**Figure 2**). No significance (P≥0.05) was seen with *P*-value of 0.2189 between the percentage of bundles and the viscosity. Although testing the different viscosities with their percentage of bundles, none of them showed significance (P≥0.05). On average, between 1.5% to 2.8% of sperm bundles were found in the semen samples after swim-up across all levels of viscosity.

### 3.2. Swim-up increases bundle formation

**Figure 3:**
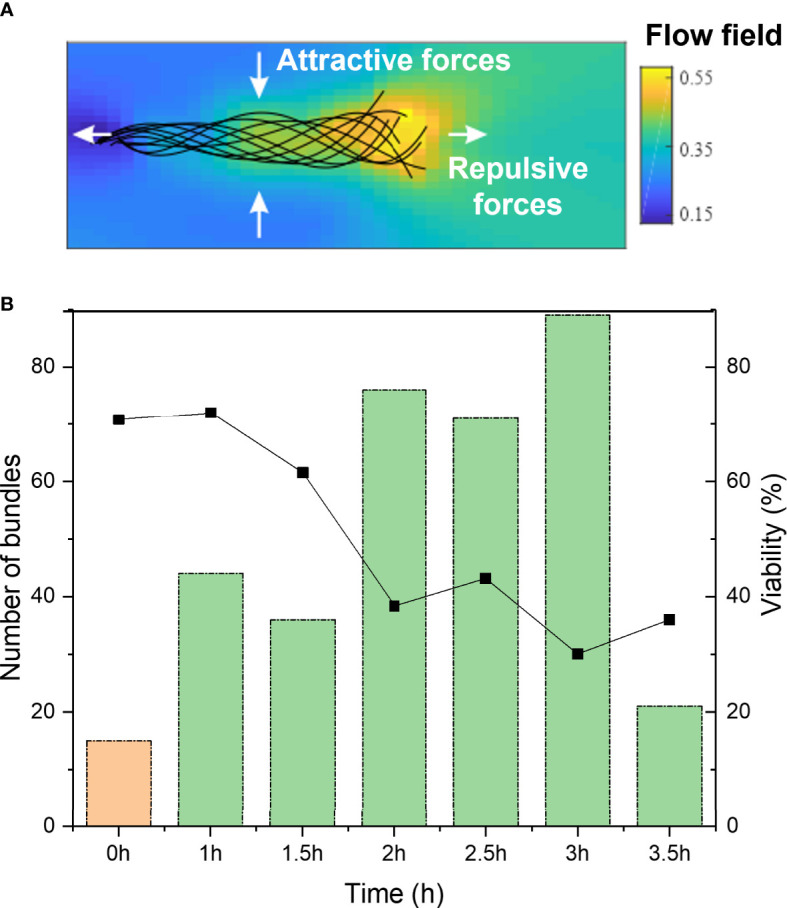
**(A)** Schematic of flow fields around a sperm cell, from head (left) to tail (right), displaying the attractive and repulsive forces. **(B)** Total number of cell bundles of bull sperm over post-thaw migration time. 0h shows before swim-up sperm at 0h time (orange), 1 to 3.5h refers to swim-up sperm (green). On the right y-axis is the overall viability plotted, obtained from fluorescent staining at the different time points.

The bundle formation was compared between samples before and after swim-up of the same semen straws. The samples before swim-up displayed a negligible amount of bundles (See **Figures 2**, **3**). After swim-up, the amount of bundles versus single cells was significantly increased from 1.5% to 2.8%. This is rooted in the fact that swim-up renders the most motile sperm and separates them from non-motile sperm. This enables more interactions between progressively moving sperm.

### 3.3 Post-thaw migration time increases probability of bundling

We also studied the influence of post-thaw migration time, meaning the time that the sperm cells were incubated and allowed to interact with each other. In **Figure 3B**, it can be seen that the percentage of bundles increases with elongated post-thaw migration time to about 3 hours (significant increase over time, *P*-value = 5.648E-11). The drop in bundle formation after three hours can be explained by the decreased overall sperm viability. This hints to the fact that the prolonged interaction time between sperm cells allows their increased interactions and bundle formation. It is known that repulsive and attractive forces exist between single sperm cells. **Figure 3A** displays a schematic of a sperm cell and its theoretical hydrodynamic flow field surrounding it. The color code shows the dimensionless fluid flow velocity. Also indicated are the repulsive and attractive forces acting on the sperm that result from the flow field. This explains how sperm cells tend to bundle with their heads joined and then synchronizing their tail beating. Besides these hydrodynamic interactions, also adhesive areas on the sperm head play a role in the sperm interaction (6).

### 3.4 Bovine serum albumin inhibits bundle occurrence

As displayed in **Figure 2B**, the occurrence of bundles is significantly reduced (*P*-value 2.00E-16) in the presence of bovine serum albumin, compared to medium without BSA (**Figure 2A**). No bundles were observed before swim-up in medium containing BSA. This means that BSA, as capacitating agent, suppresses adhesion between sperm and thus inhibits bundle formation.

### 3.5 Effect of viscosity and cell number per bundle on velocity

**Figure 4:**
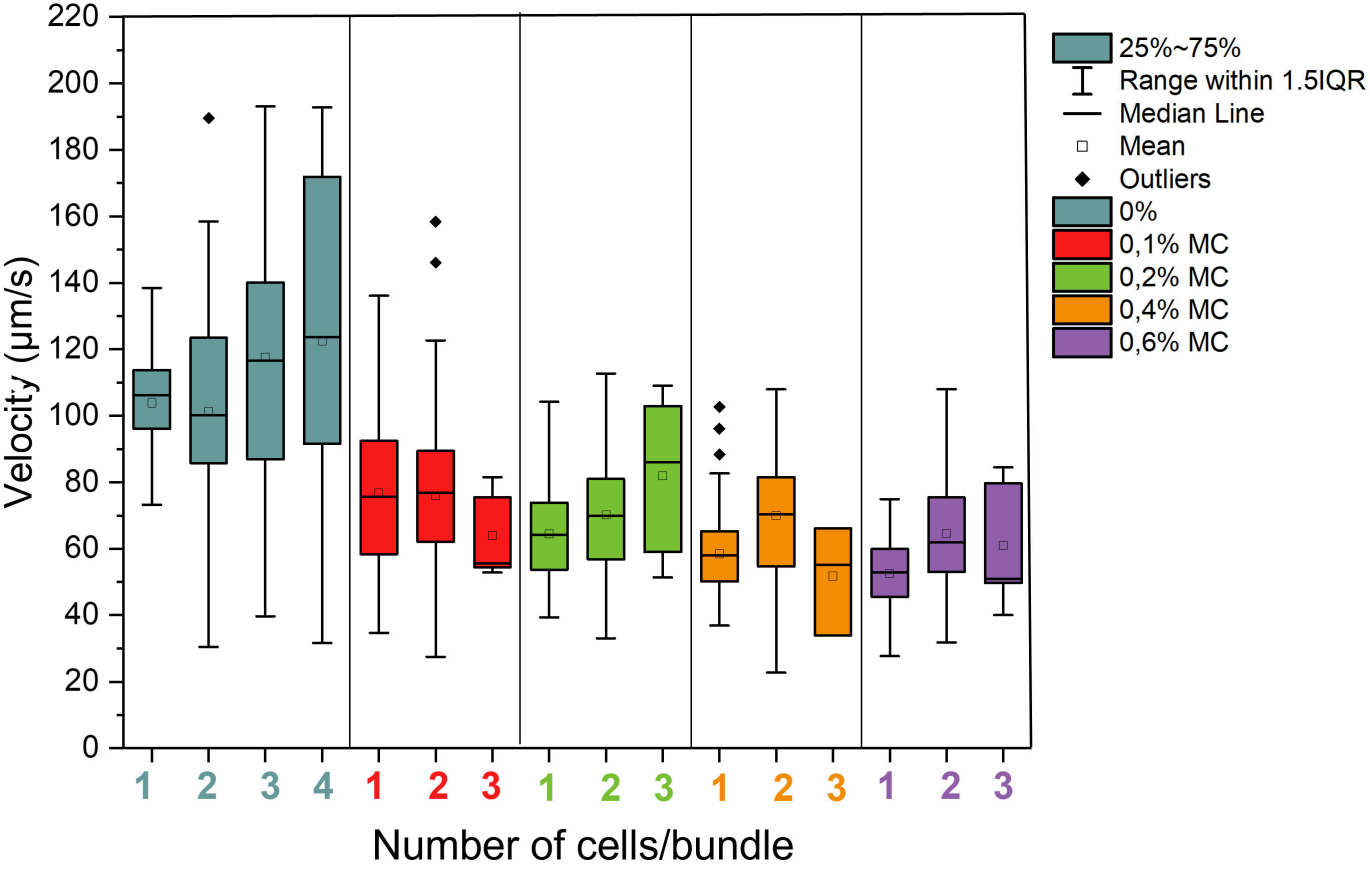
Velocities of bundles of bull sperm depending on cell number per bundle in different levels of viscosity. A total of 136 sperm cells were studied at 0%, 116 at 0.1%, 138 at 0.2%. 137 at 0.4% and 135 at 0.6%, a total amount of 662 sperm cells were studied.

The velocities of the sperm bundles was also analyzed and compared to single sperm cells in the same conditions (**Figure 4**). In general, the velocities of sperm are reduced in elevated viscosities compared to medium without methyl cellulose. With increasing number of cells in the bundle, an increase in velocity was observed in case of low viscosity medium (0% MC) when 3 or 4 cells were contained in the bundle. Paired swimmers were on average slower than single sperm in pure DMEM medium. In 0.2%, 0.4% and 0.6% MC, the velocity of paired sperm increased significantly compared to single sperm (*P*-value of 0.012 in 0.2% comparing one cell and three cells bundle, 6.7E-05 in 0.4% comparing one cell and two cells bundle and 1.1E-04 in 0.6% comparing one cell and two cells bundles). Here, the effect of viscosity can be seen which enables an improved hydrodynamic interaction between the sperm and thus improved swimming speed in high viscosity.

### 3.6 Non-capacitated cells bundle more frequently

**Figure 5:**
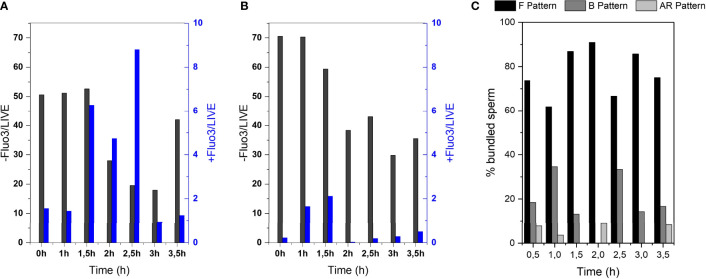
Investigation of amount of capacitated sperm over post-thawing incubation time. Flow cytometry results of Fluo-3 stained bull sperm at different post-thaw incubation times after swim-up in **(A)** DMEM **(B)** DMEM + BSA. **(C)** CTC assay results of sperm bundles showing the % of bundled sperm displaying the F pattern (non-capacitated), B pattern (capacitated, acrosome intact) or AR pattern (acrosome reacted).

In order to investigate if the capacitation state of the cells influences the bundle formation, we performed flow cytometry of sperm cells that were stained to mark their capacitation state. Sperm cells were incubated with Fluo-3 and PI. This dye is fluorescent in the presence of calcium ions. Since sperm capacitation is linked to an increased intracellular calcium concentration, this dye can be used to measure the capacitation state of the cells (34). **Figure 5** displays that the percentage of non-capacitated cells in the black data set (Fluo-/Live) was higher in DMEM(A) than in DMEM with BSA (B). In contrast, it also displays that the percentage of capacitated cells in the blue data set (FLUO+/Live) was higher in DMEM with BSA (B) than in DMEM (A). Focusing on live sperm cells with calcium efflux in both media (**Figures 5A, B**, (FLUO+/Live)), there is a significant increase (*P*-value 0.0354) in DMEM + BSA medium comparing with DMEM one, as BSA induces Ca^2+^ influx in sperm cells, seeing an increase of capacitated cells by the time. Intracellular calcium levels slightly increased in non-capacitating sperm medium (**Figure 5A**). This can be explained as cryopreservation can alter sperm plasma membrane leading to a premature calcium influx and sperm capacitation (35).

CTC stains were performed to evaluate specifically the sperm that formed bundles (**Figure 5C**). With the help of this stain, sperm cells can be categorized into non-capacitated (F pattern), capacitated (F pattern) and acrosome reacted (AR pattern). It can be seen that the majority of sperm that formed bundles were non-capacitated.

## 4 Discussion

The viscosity of the medium does not seem to enhance or avoid bundle formation, although further investigation combined with hydrodynamic modelling might be helpful to elucidate those interactions. In both media (with and without bovine serum albumin), we can see a significant difference (P ≤ 0.05) between the percentage of bundles formed before and after swim-up, obtaining a *P*-value of 9.51E-04 with DMEM + BSA and a ≤ 2.00E-16 withDMEM. From our investigations, it can be concluded that the post-thaw migration time of cryopreserved bull sperm allows increased bundling over time. Furthermore, the swim-up procedure significantly increases the amount of bundled sperm. This gives implications with clinical relevance about the swim-up of cryopreserved sperm.

From analysis of the capacitation state of the sperm cells by the help of flow cytometry of Fluo-3 stained cells and CTC assay of sperm bundles, it seems that bundles occur more frequently in the non-capacitated fraction of sperm cells. For proving a clear connection, however, further investigations are required. One can note that consistently over 60% of bundled sperm showed the F pattern, meaning they were non-capacitated (**Figure 5C**). Further, the fact that capacitation-inducing medium (DMEM and BSA) suppresses the bundle formation (**Figure 2A**) is another indicator that sperm cooperative behaviour occurs preferentially in non-capacitating conditions. The more frequent bundling of non-capacitated sperm goes in line with previous description by Moore et al. (10), in which the eventual dispersal of sperm trains in mice was associated with most of the spermatozoa undergoing acrosome reaction.

Overall, it seems that the bundle formation is mostly related to increased interaction time between sperm, which allows accumulation of bundles over post-thaw migration time. At the same time, the overall motility decreases over time, resulting in the maximum bundle appearance between 1.5 to 3 hours. Further, spermatozoa seem to have a higher tendency to bundle in non-capacitating conditions. Since we only investigated cryopreserved bull semen in this study, we cannot evaluate the influence of seminal plasma proteins on the sperm bundling. It is known from previous studies that seminal plasma components modulate cell-cell adhesion (36–38). Specifically, they mediate the adhesion of sperm cells to the oviduct reservoir (notably, spermadhesins in pigs and cattle). Yet, these proteins do not truly inhibit protein-protein interactions, but keep sperm in an uncapacitated stage (39). These components delay or prevent sperm capacitation. Sperm capacitation, in turn, goes in hand with making cell membranes more fusogenic by removing cholesterol from the plasma membrane and outer acrosome membranes can fuse (40, 41). This facilitates the attachment of sperm cells to each other, interact and agglutinate. We have observed strong sperm bundling in fresh boar semen. But this remains subject of further studies.

In conclusion, sperm bundling is a cooperative behaviour observed in bull sperm, which leads to formation of sperm pairs and less frequently also to groups of 3 or more sperm. Swim-up enhances this cooperative behaviour probably due to the applied selection process towards the most motile sperm. This cooperation seems to apply mostly to non-capacitated, migrating sperm. We found no influence of viscosity on bundle formation. This strengthens the hypothesis that bundling is a mechanism by which sperm assist each other to overcome large swimming distances before approaching the egg. Upon arrival at the fertilization site, capacitation process is required for proper maturation of the cells and fusion with the oocyte. With that, and concluding from our analysis, cooperative bundling is probably suppressed in this final stage.

The bundling of sperm investigated in this study gives implications for sperm competition and cooperativity. Sperm bundling does not relate directly to sperm function, but rather to the sperm´s ability to perform smart collective behaviour. This study gives a hint that sperm use cooperative behavior to overcome long migration distances rather far from the egg. It is an interesting aspect particularly for evolutionary sperm biology, to understand how this sperm bundling affects sperm competition. Further, from a biophysical view point, it will be interesting to study how the flagella of the bundles are synchronized to achieve faster swimming speed. In this study, we focused on the average overall swimming speed of the bundles. Detailed flagella waveform analysis will reveal in future studies how the synchronization of the flagella plays a role in the resulting bundle speed. Further, it will be interesting to understand if and how the sperm cells use this cooperative behavior to potentially save energy along the way. Sperm cells have a limited lifetime and energy reservoir. Most of their energy consumption goes into motility, so it is obvious that swimming more efficiently by cooperation will give those cells an advantage over single cells. In conclusion, sperm cooperative behavior is an important aspect to study sperm migration and might also give future indications for reasons of infertility, together with understanding metabolic and molecular mechanisms. In turn, this will give insight into sperm selection, extending fertility and controlling the capacitation state of sperm as tool in reproductive biotechnologies.

## Data availability statement

The original contributions presented in the study are included in the article/**Supplementary Material**. Further inquiries can be directed to the corresponding author.

## Author contributions

VM conceived the project, planned the study, performed experimental work and supervision. PM conducted experimental work, data analysis and statistical treatment. MY supervised the work, supported in experimental work and gave input throughout the study. LZ and ZF supported sperm analysis with ONGO device. CH provided bull semen. All authors contributed to the article revision. All authors contributed to the article and approved the submitted version.

## Funding

VM acknowledges funding from the “la Caixa” Foundation (ID 100010434) and from the European Union’s Horizon 2020 research and innovation programme under the Marie Skłodowska-Curie grant agreement No LCF/BQ/PI21/11830003. The authors also acknowledge the support from the Ministry of Science and Innovation, Spain (Grants: AGL2017-88329-R and PID2020-113320RB-I00), the Catalan Agency for Management of University and Research Grants, Regional Government of Catalonia, Spain (Grant: 2017-SGR-1229), and the Catalan Institution for Research and Advanced Studies (ICREA). ZF and LZ acknowledge funding from the National Research Development & Innovation Office (grant number: TKP2021-EGA-42).

## Acknowledgments

VM thanks Samuel Sanchez and the Smart Nanobiodevices lab for providing lab space and equipment to conduct the study and ASEAVA and ASCOL, for providing semen straws.

## Conflict of interest

Authors ZF and LZ were employed by ONGO Vettech Ltd.

The remaining authors declare that the research was conducted in the absence of any commercial or financial relationships that could be construed as a potential conflict of interest.

## Publisher’s note

All claims expressed in this article are solely those of the authors and do not necessarily represent those of their affiliated organizations, or those of the publisher, the editors and the reviewers. Any product that may be evaluated in this article, or claim that may be made by its manufacturer, is not guaranteed or endorsed by the publisher.
