# Supplementary material for: Bundle formation of sperm: Influence of environmental factors
Source: Front Endocrinol (Lausanne). 2022 Oct 10;13:957684. doi: 10.3389/fendo.2022.957684 (PMC9591104; doi:10.3389/fendo.2022.957684)
Supplement: Supplementary file 1 [file DataSheet_1.pdf]

Supplementary Material

1 SUPPLEMENTARY VIDEOS

Video 1: Two sperm bundle of 2 cells in 0.1% methyl cellulose. Recorded in real time with 100 frames per second in phase contrast. Scale bar 20 μ m.

Video 2: Sperm bundle with 3 cells. Recorded with 100fps, playing 5x slower than real time (20fps). Scale bar 20 μ m.

Video 3: Sperm bundle with 4 cells. Recorded with 100fps, Video playing 5x slower than real time (20fps).

Video 4: Sperm bundle of 5 cells in 0.4% methyl cellulose. Recorded in real time with 100 frames per second in phase contrast, played 5x slower than real time (20fps). Scale bar 20 μ m.

2 SUPPLEMENTARY TABLES AND FIGURES

2.1 Figures

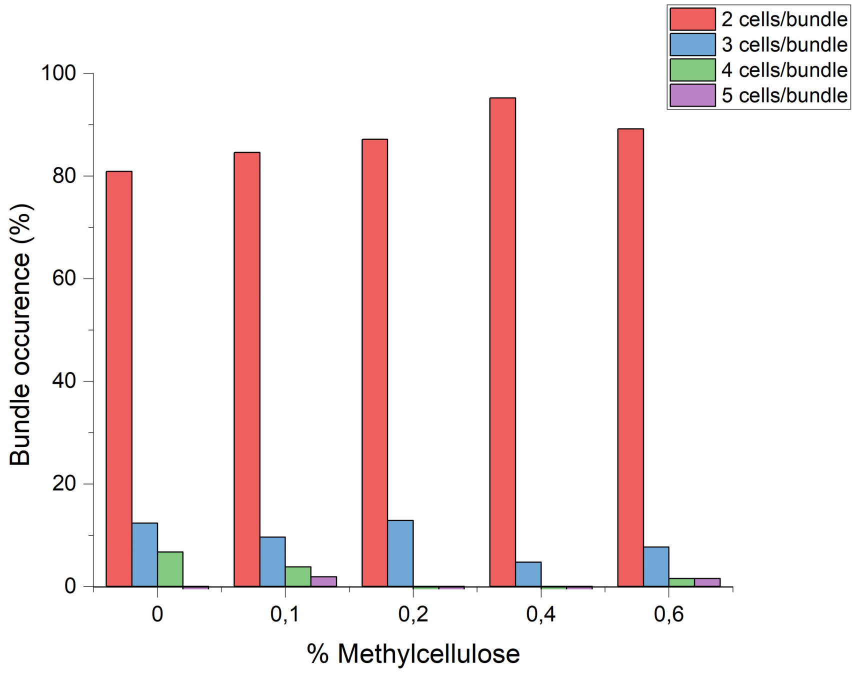

Figure S1. Number of sperm cells per bundle depending on media viscosity.

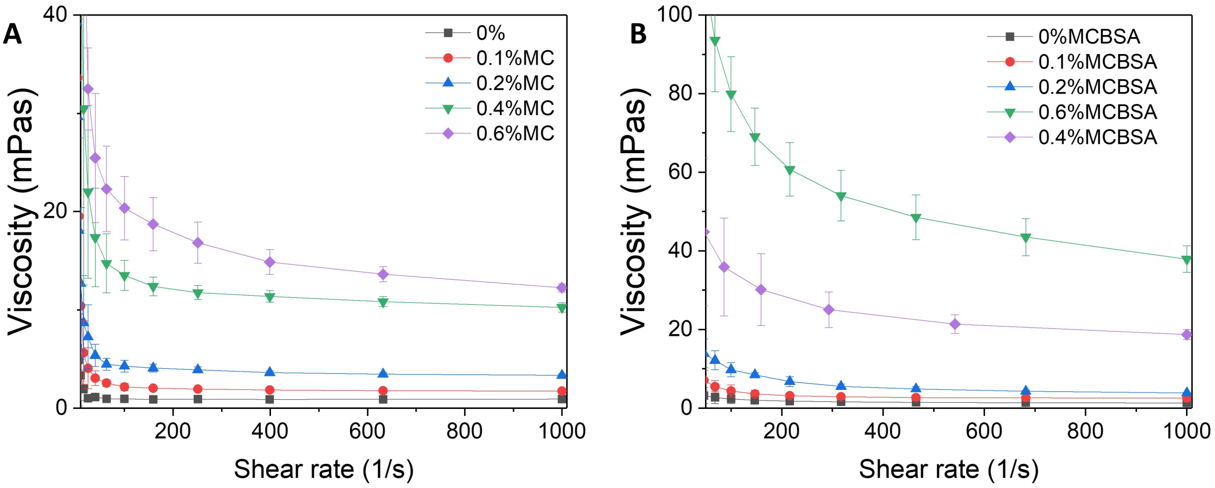

Figure S2. Viscosity over shear rate of (A) DMEM without and (B) DMEM with BSA, both with 0 - 0.6% concentrations of methyl cellulose).

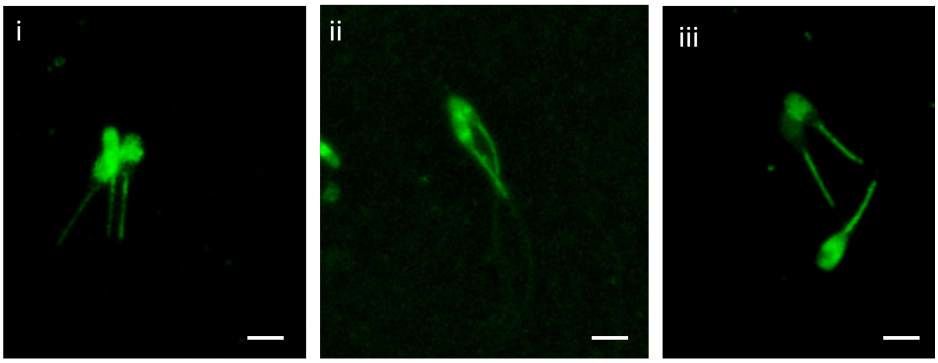

Figure S3. CTC stain of bundles sperm showing (i)F-pattern of non-capacitated sperm, (ii) B pattern of capacitated, acrosome-intact sperm and (iii) AR pattern of acrosome-reacted(left) and B pattern (right) capacitated sperm.

**Factor**	**P-Value**
**Number bundles swim-up vs. no swim-up (DMEM)**	2,00E-16
**Number bundles swim-up vs. no swim-Up (DMEM+BSA)**	9,51E-05
**Number bundles swim-up (DMEM vs. DMEM + BSA)**	2.2E-16

Table S1. Statistics done by a Kruskal-Wallis test.

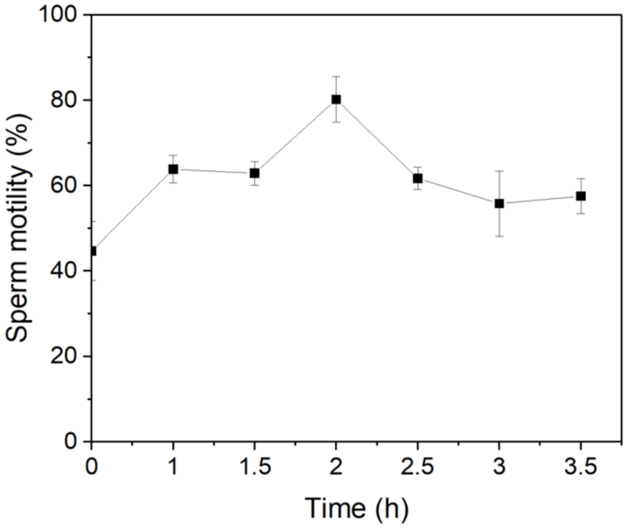

Figure S4. Overall motility during swim-up process of bull sperm monitored over time with ONGO semen analyzer in DMEM medium, 0% Methyl cellulose.

**Factor**	**P-Value**
**DMEM Bundles - Viscosities**	0.219
**0% - 0.1%**	0.383
**0% - 0.2%**	0.228
**0% - 0.4%**	0.634
**0% - 0.6%**	0.634
**0.1% - 0.2%**	0.204
**0.1% - 0.4%**	0.022
**0.1% - 0.6%**	0.144
**0.2% - 0.4%**	0.228
**0.2% - 0.6%**	0.353
**0.4% - 0.6%**	0.634
**DMEM+BSA Bundles - Viscosities**	0.012
**0% - 0.1%**	0.186
**0% - 0.2%**	0.485
**0% - 0.4%**	0.428
**0% - 0.6%**	0.067
**0.1% - 0.2%**	0.067
**0.1% - 0.4%**	0.485
**0.1% - 0.6%**	0.200
**0.2% - 0.4%**	0.165
**0.2% - 0.6%**	0.030
**0.4% - 0.6%**	0.140

Table S2. Statistics done using Kruskal-Wallis test, tests to check in-between groups are done with Wilcox test.

**Factor**	**P-Value**
**DMEM + Swim-up + Time**	5.648E-11
**1h – 1.5h**	0.213
**1h – 2h**	0.027
**1h – 2.5h**	0.996
**1h – 3h**	4.7E-04
**1h – 3.5h**	1.8E-04
**1.5h – 2h**	0.636
**1.5h – 2.5h**	0.069
**1.5h – 3h**	1.2E-04
**1.5h – 3.5h**	3.3E-04
**2h – 2.5h**	0.002
**2h – 3h**	2.9E-07
**2h – 3.5h**	2.4E-05
**2.5h – 3h**	1.8E-04
**3h – 3.5h**	0.046

Table S3. Statistics done using Kruskal-Wallis test.

**Factor**	**P-Value**
**Number cells vs. Velocity (0%)**	0.481
**One – Two**	0.650
**One – Three**	0.560
**One – Four**	0.650
**Two – Three**	0.560
**Two – Four**	0.560
**Three - Four**	0.860
**Number cells vs. Velocity (0.1%)**	0.475
**One – Two**	0.910
**One – Three**	0.390
**Two – Three**	0.390
**Number cells vs. Velocity (0.2%)**	7.4E-03
**One – Two**	0.057
**One – Three**	0.012
**Two – Three**	0.057
**Number cells vs. Velocity (0.4%)**	5.48E-05
**One – Two**	6.7E-05
**One – Three**	0.062
**Two – Three**	0.446
**Number cells vs. Velocity (0.6%)**	2.1E-04
**One – Two**	1.1E-04
**One – Three**	0.567
**Two – Three**	0.567

Table S4. Statistics done using Kruskal-Wallis test, tests to check in-between groups are done with Wilcox test.

**Factor**	**P-Value**
**Fluo+/Live with DMEM and Fluo+/Live DMEM+BSA**	0.0354

Table S5. Statistics done using ANOVA test.
